# Anti-Tumor Effects after Adoptive Transfer of IL-12 Transposon-Modified Murine Splenocytes in the OT-I-Melanoma Mouse Model

**DOI:** 10.1371/journal.pone.0140744

**Published:** 2015-10-16

**Authors:** Daniel L. Galvan, Richard T. O’Neil, Aaron E. Foster, Leslie Huye, Adham Bear, Cliona M. Rooney, Matthew H. Wilson

**Affiliations:** 1 Center for Cell and Gene Therapy, Baylor College of Medicine, Houston, Texas, United States of America; 2 Department of Pediatrics, Baylor College of Medicine, Houston, Texas, United States of America; 3 Tennessee Valley Health Care, Department of Veterans Affairs, Nashville, Tennessee, United States of America; 4 Department of Medicine, Vanderbilt University School of Medicine, Nashville, Tennessee, United States of America; Ohio State University, UNITED STATES

## Abstract

Adoptive transfer of gene modified T cells provides possible immunotherapy for patients with cancers refractory to other treatments. We have previously used the non-viral *piggyBac* transposon system to gene modify human T cells for potential immunotherapy. However, these previous studies utilized adoptive transfer of modified human T cells to target cancer xenografts in highly immunodeficient (NOD-SCID) mice that do not recapitulate an intact immune system. Currently, only viral vectors have shown efficacy in permanently gene-modifying mouse T cells for immunotherapy applications. Therefore, we sought to determine if *piggyBac* could effectively gene modify mouse T cells to target cancer cells in a mouse cancer model. We first demonstrated that we could gene modify cells to express murine interleukin-12 (p35/p40 mIL-12), a transgene with proven efficacy in melanoma immunotherapy. The OT-I melanoma mouse model provides a well-established T cell mediated immune response to ovalbumin (OVA) positive B16 melanoma cells. B16/OVA melanoma cells were implanted in wild type C57Bl6 mice. Mouse splenocytes were isolated from C57Bl6 OT-I mice and were gene modified using *piggyBac* to express luciferase. Adoptive transfer of luciferase-modified OT-I splenocytes demonstrated homing to B16/OVA melanoma tumors *in vivo*. We next gene-modified OT-I cells to express mIL-12. Adoptive transfer of mIL-12-modified mouse OT-I splenocytes delayed B16/OVA melanoma tumor growth *in vivo* compared to control OT-I splenocytes and improved mouse survival. Our results demonstrate that the *piggyBac* transposon system can be used to gene modify splenocytes and mouse T cells for evaluating adoptive immunotherapy strategies in immunocompetent mouse tumor models that may more directly mimic immunotherapy applications in humans.

## Introduction

Adoptive transfer of gene modified T cells has been used successfully for immunotherapy of cancer in humans [1, 2]. Viral vectors, most commonly retroviruses, have been used to gene modify T cells for adoptive immunotherapy [3]. Non-viral transposons provide an alternative methodology for permanent genetic modification of human T lymphocytes. The *Sleeping Beauty* transposon system is currently approved for a clinical trial targeting CD-19 positive B cell malignancies [4–6]. Transposons have several advantages over viral vectors as they are relatively inexpensive, enabling more nimble evaluation of different modifying constructs, and have a larger capacity than retro- or lentiviral vectors which may promote more widespread use [7].

The *piggyBac* transposon system has also been evaluated for immunotherapy applications. *piggyBac* has a few advantages including high activity [8, 9], large cargo capacity [10], the ability to co-deliver multiple genes [11], and excision without genome mutation [12, 13]. We have demonstrated efficient long-term gene-modification of human T lymphocytes [14]. *piggyBac*-modified human T cells have demonstrated anti-tumor activity against tumor cells *in vitro* [15, 16] and against tumor xenografts in NOD-SCID mice *in vivo* [17].

The most common approach for pre-clinical *in vivo* testing of anti-tumor activity of adoptively transferred human T cells involves tumor xenograft implantation in highly immunodeficient mice. These models have the advantage of testing the ability of human T cells to target and kill cancer xenograft cells *in vivo* in an animal model. However, a major disadvantage is the lack of an immune system that can better mimic the true setting of a cancer patient. This is particularly relevant when testing constructs developed to counteract immune evasion strategies. Adoptive transfer of gene-modified murine T cells may provide important insights when they are adoptively transferred into syngeneic immunocompetent animals. Murine T cells are amenable to transfection with DNA plasmids [18]. Cut-and-paste DNA transposons offer permanent integration of delivered DNA cargo after transfection. However, *piggyBac* transposon modification of mouse T cells has not been reported.

The OT-I mouse model in combination with ovalbumin-modified B16 melanoma cells (B16/OVA) has been used to test immunotherapy of melanoma *in vivo* [19]. The OT-I T cells carry transgenic inserts for the *Tcra-V2* and *Tcrb-V5* genes designed to recognize an ovalbumin epitope, thereby directing the cells to the tumor and eliciting a CD8 positive T cells response to tumor cells expressing ovalbumin antigens [20]. B16/OVA melanoma cells have been gene modified to express ovalbumin, and can be implanted into mice to generate tumors [21]. We chose the OT-I/B16 melanoma model to determine if transgene modification of splenocytes, including mouse T cells, might improve anti-tumor activity *in vivo*. Autologous peripheral blood mononuclear cells gene modified to express melanoma antigen-specific T cell receptors have mediated tumor regression in melanoma patients [22–24].

IL-12 is a pleiotropic cytokine bridging innate and adaptive immunity and creating its appeal in tumor immunotherapy. Severe toxicities associated with the systemic use of IL-12 have led researchers to evaluate for safer and effective results with directed delivery. IL-12 exerts its anti-tumor activity in part by directly enhancing the cytotoxic activity of T lymphocytes and in part by acting on local professional antigen presenting cells, reversing their immunosuppressive activity [25, 26]. Local delivery has been accomplished by gene modified immune cell delivery [27], modified fibroblasts [28, 29], and direct modification of the tumor [30]. It has also been suggested that IL-12 can enhance antitumor radiotherapy while diminishing acute radiation injury [31, 32]. After early results raising safety concerns with the therapeutic use of IL-12, interest has resurfaced with investigation of local delivery schemes.

Zhang and colleagues have previously demonstrated improvement of adoptive T cell therapy for melanoma using the B16 mouse model and inducible IL-12 expressed from a retroviral vector [33]. Given the expense of retroviral vector production for human application and the disadvantages of using tumor xenografts in NOD-SCID mice, we sought to determine if *piggyBac* could be used for non-viral gene modification of mouse T lymphocytes to express IL-12 and improve adoptive transfer mediated anti-tumor activity in the mouse B16 melanoma tumor model.

## Results

### Engineering cells using *piggyBac* for stable IL-12 production

**Fig 1:**
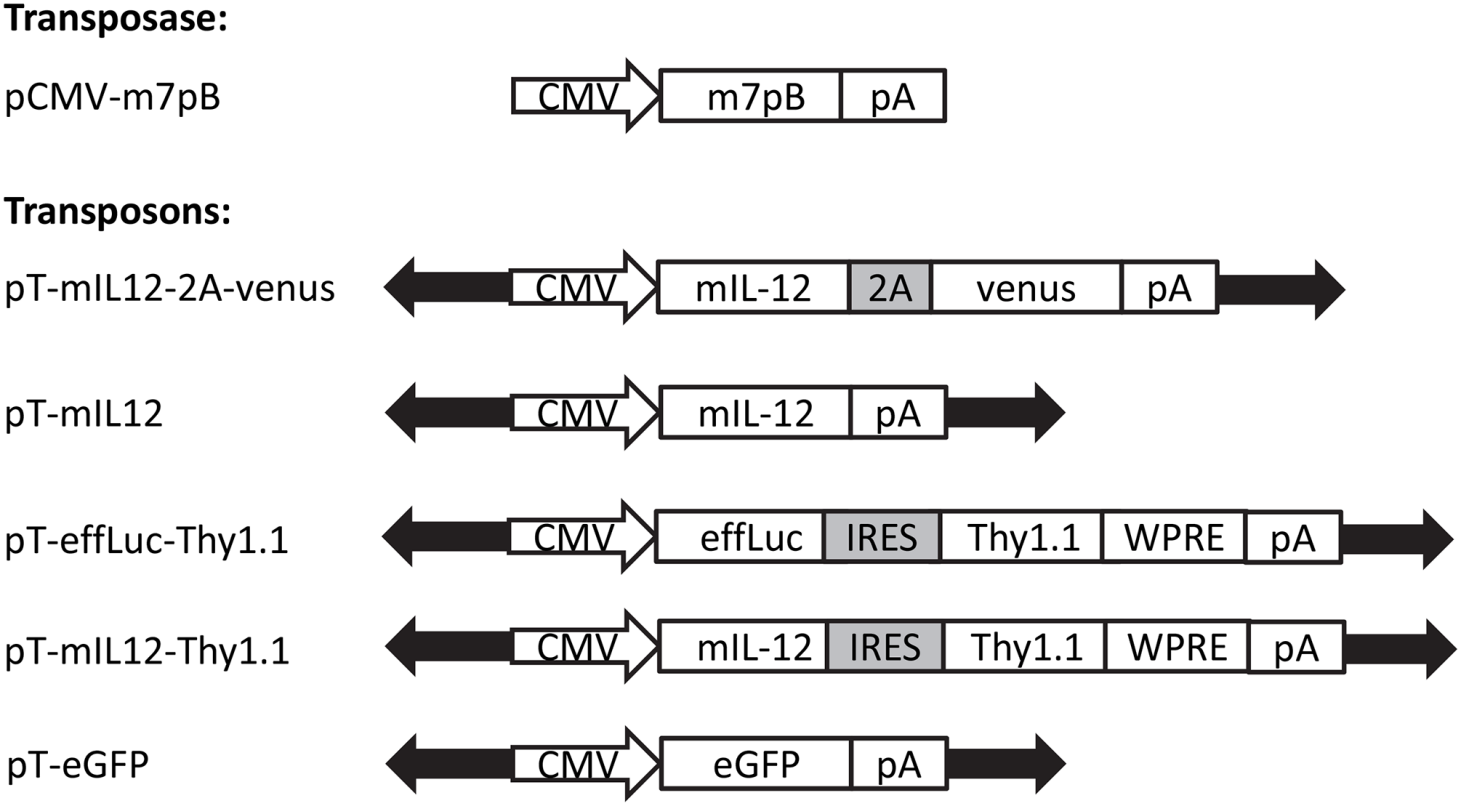
Vector schematics. The hyperactive (m7pB) *piggyBac* transposase was used in combination with various transposons for mIL-12 and/or reporter gene (venus or luciferase) expression *in vitro* or *in vivo*. CMV, cytomegalovirus immediate early enhancer/promoter; *piggyBac*, transposase; pA, SV40 polyadenylation signal; mIL-12, murine IL-12; 2A, 2A sequence; venus, reporter gene; effLuc, enhanced luciferase reporter gene; stop, stop codon; IRES, internal ribosomal entry site; Thy1.1, mouse Thy1.1 antigen; WPRE, woodchuck hepatitis post-transcriptional regulatory element; eGFP, enhanced green fluorescent protein.

**Fig 2:**
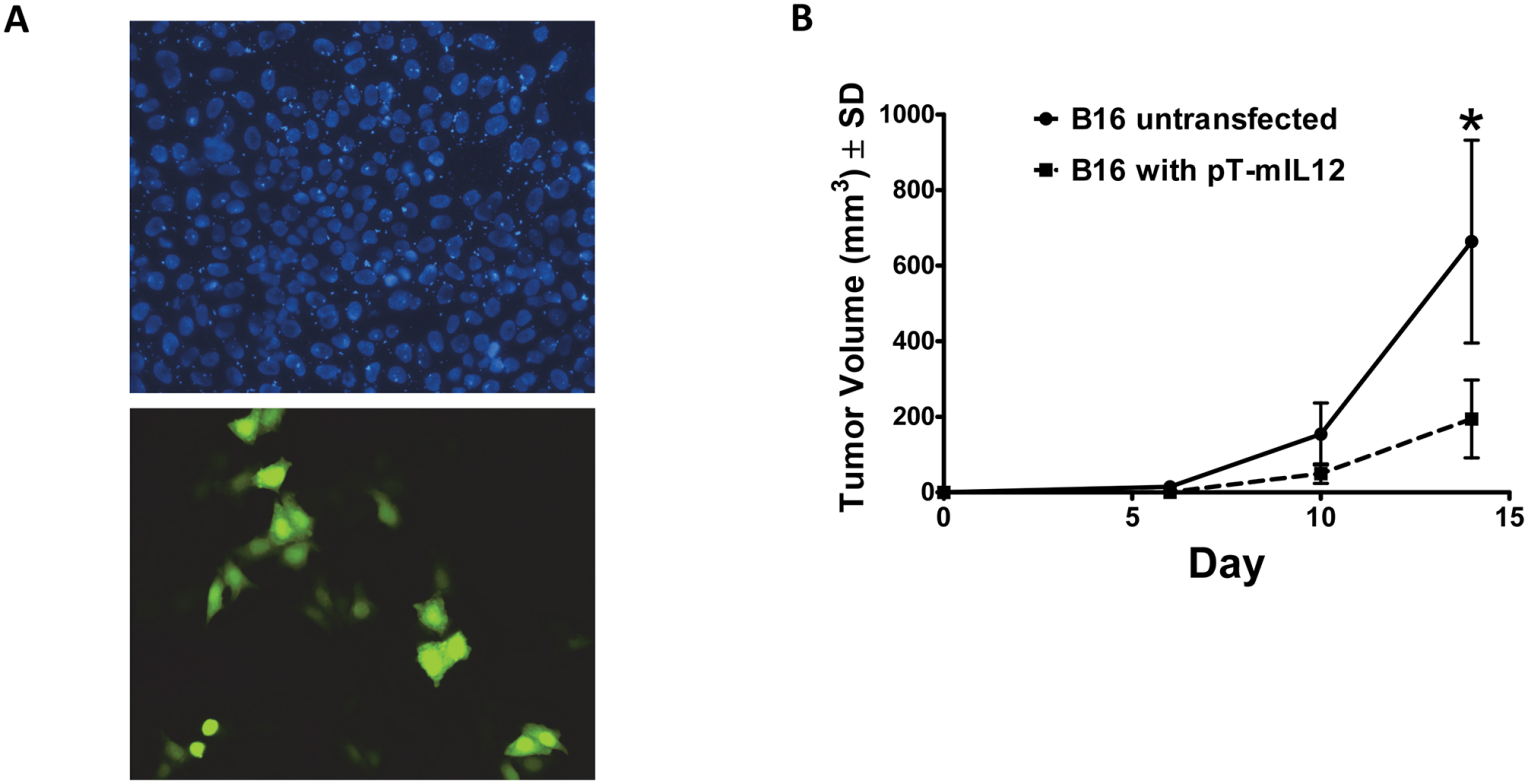
Functional expression of IL-12 from a *piggyBac* transposon. **A**, HeLa cells were transfected with the pT-IL12-2A-venus transposon. DAPI (4',6-diamidino-2-phenylindole) stain was utilized to visualize cell nuclei (**top**) and immunofluorescence of the venus reporter gene was used to visualize protein expression (**bottom**). Shown is a representative of 3 independent experiments. Culture media from these cells was analyzed for mIL-12 production resulting in 31 ± 5pg/μl of mIL-12 (N = 3, ± SEM). **B**, B16 melanoma cells were stably transfected with pT-mIL12 in the presence of pCMV-m7pB. 5 X 10^5^ B16 cells were implanted into mice on the hind quarter. The ability of mIL-12 expressing B16 cells to affect the growth of contralaterally implanted B16 cells was compared to that of untransfected cells. mIL-12 expressing B16 cells inhibited the contralateral growth of B16 cell *in vivo*.

The *piggyBac* vectors we used in our studies are shown in Fig 1. We engineered three *piggyBac* transposon vectors to express mIL-12 (p35/p40 IL-12): one followed by the venus fluorescent reporter after a 2A cleavage sequence, one followed by the Thy1.1 antigen, and one with mIL-12 alone. To validate IL-12 production, HeLa cells (1x10^6^) were transiently transfected with pT-mIL12-2A-Venus and subjected to fluorescent microscopy for detection of the Venus reporter gene (Fig 2A). Media was collected prior to imaging at 24 hours post-transfection and mIL-12 concentration was measured using ELISA. Secreted mIL-12 was detected at a concentration of 31 ± 5pg/μl (N = 3, ± SEM) (S1 Fig). Therefore, *piggyBac* could be engineered to produce mIL-12 which could be secreted and detected.

To determine if the IL-12 produced was biologically active, we tested its ability to prevent tumor growth when expressed from the tumor cells. We transplanted 5 X 10^5^ B16 melanoma cells stably transfected with pCMV-PB and pT-IL12 into C57Bl6 mice and compared their growth to control unmodified B16 melanoma cells. IL-12 producing tumors failed to grow and also slowed the growth of contralateral unmodified tumor cell transplants *in vivo* (Fig 2B), compared to unmodified control tumor cells alone, implying the induction of an endogenous immune response. Therefore, our mIL-12 producing *piggyBac* vector elicited anti-melanoma tumor activity *in vivo* when expressing mIL-12 from melanoma cells.

### *piggyBac*-mediated gene modification of mouse T cells

**Fig 3:**
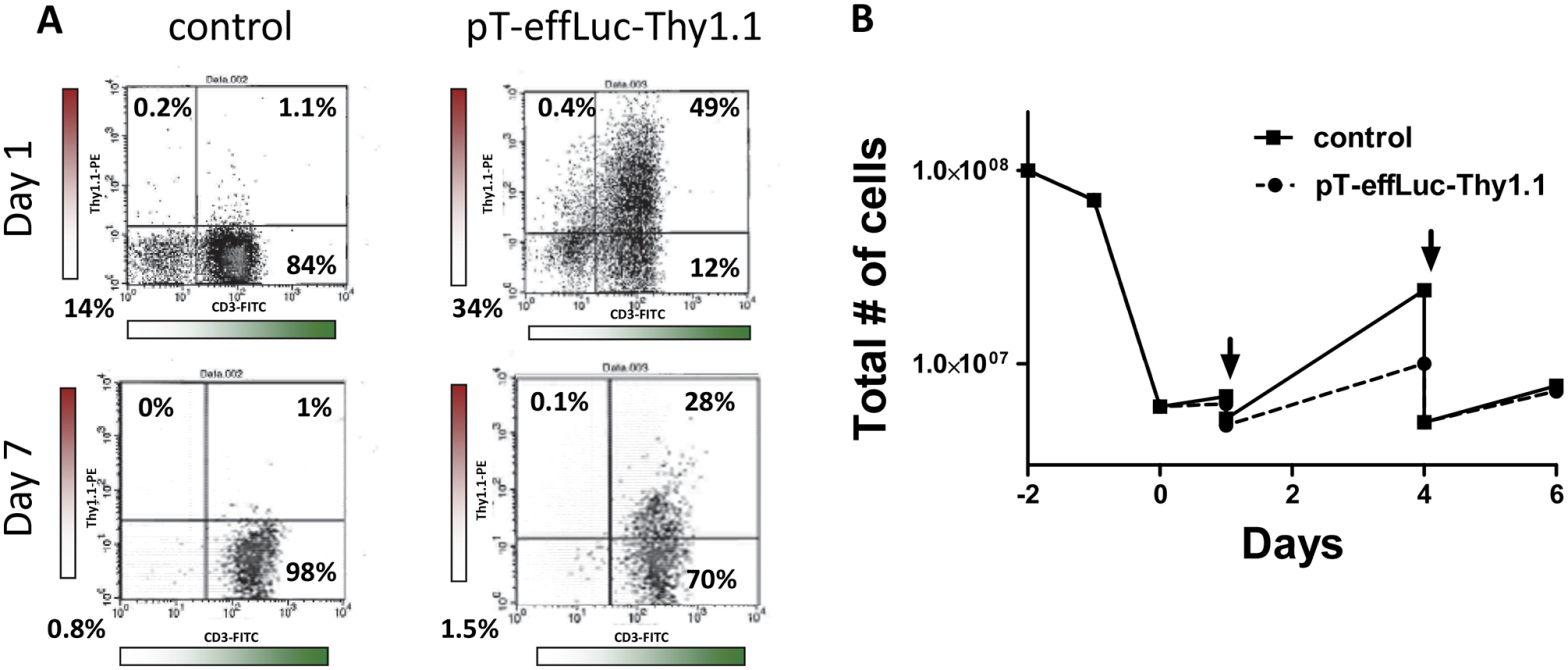
*piggyBac* transposon modification of mouse splenocytes. **A**, Splenocytes were transfected with pCMV-m7pB and pT-effLuc-Thy1.1 using the Neon transfection system and transfection efficiency was evaluated via flow cytometry using antibodies directed against the Thy1.1 antigen at day 1 and day 7 post transfection. Shown is a representative of 3 independent experiments. **B**, mouse splenocytes could be cultured short term exhibiting cell growth. Shown is a representative of 3 independent experiments. Arrows indicate a split to 5 X 10^6^ cells for continued growth.

To determine the efficiency of *piggyBac* for the gene modification of murine T cells, we created a *piggyBac* vector expressing the luciferase reporter and Thy1.1 (Fig 1). Mouse splenocytes were transfected with pT-effLuc-Thy1.1 and pCMV-PB, then stimulated with concanavalin A, and transfection efficiency was quantitated by flow cytometry using an antibody to Thy1.1. From an initial transfection efficiency of 49% (± 5%, N = 3, SEM), 28% (± 4%, N = 3, SEM) of Thy1.1 and CD3 positive cells persisted on day 7 (Fig 3A). Stably transfected mouse T cells exhibited growth in short-term culture *in vitro* (Fig 3B).

### *piggyBac*-modified mouse splenocytes home to tumor sites *in vivo*

**Fig 4:**
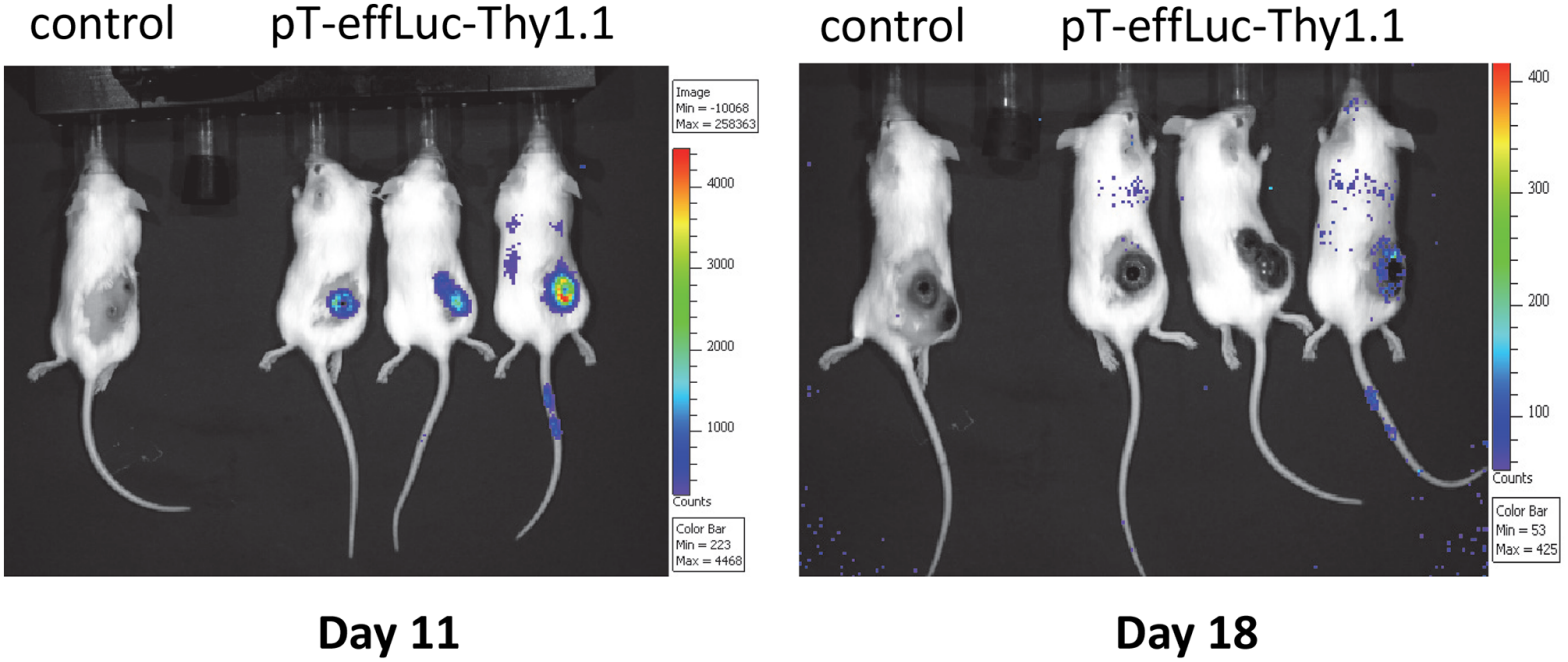
Homing of *piggyBac*-modified mouse splenocytes to tumor sites *in vivo*. OT-I mouse splenocytes were transfected with pCMV-m7pB and pT-effLuc-Thy1.1. 5 X 10^5^ B16/OVA cells into the flank of C57Bl6 mice (day –8). *piggyBac*-modified splenocytes were adoptively transferred via tail vein injection on day 0 and day +8. Localization of infused splenocytes was visualized via *in vivo* imaging of luciferase expression on day +11. Show are 3 of 6 representative animals.

The Thy1.1 transgene permitted detection of transfection efficiency using flow cytometry, whereas the luciferase transgene (Fig 1) permitted *in vivo* imaging after adoptive transfer of transfected splenocytes. We implanted 5 X 10^5^ B16/OVA cells into the flank of wild-type C57Bl6 mice (day –8). Splenocytes were isolated from OT-I C57Bl6 mice and transfected with pCMV-PB and pT-effLuc-Thy1.1. Twenty four hours after transfection, activated transgenic OT-I splenocytes were adoptively transferred via tail vein injection on day 0 and day +8. We performed *in vivo* imaging of luciferase expression to evaluate localization of stably transfected OT-I mouse T cells on day +11. We observed localization of transgenic OT-I splenocytes at sites of tumor eleven days post adoptive transfer (Fig 4). Transgene-modified cells exhibited concentrated localization at the melanoma tumor transplant site. These results confirmed that *piggyBac*-transgene modified OT-1 splenocytes could be adoptively transferred, imaged with *in vivo* imaging, and confirmed homing to tumor sites *in vivo*. However, cells exhibited limited persistence *in vivo* based on imaging at day 18 post transfer (Fig 4).

### IL-12 modified cells inhibit melanoma tumor growth and improve survival *in vivo*

**Fig 5:**
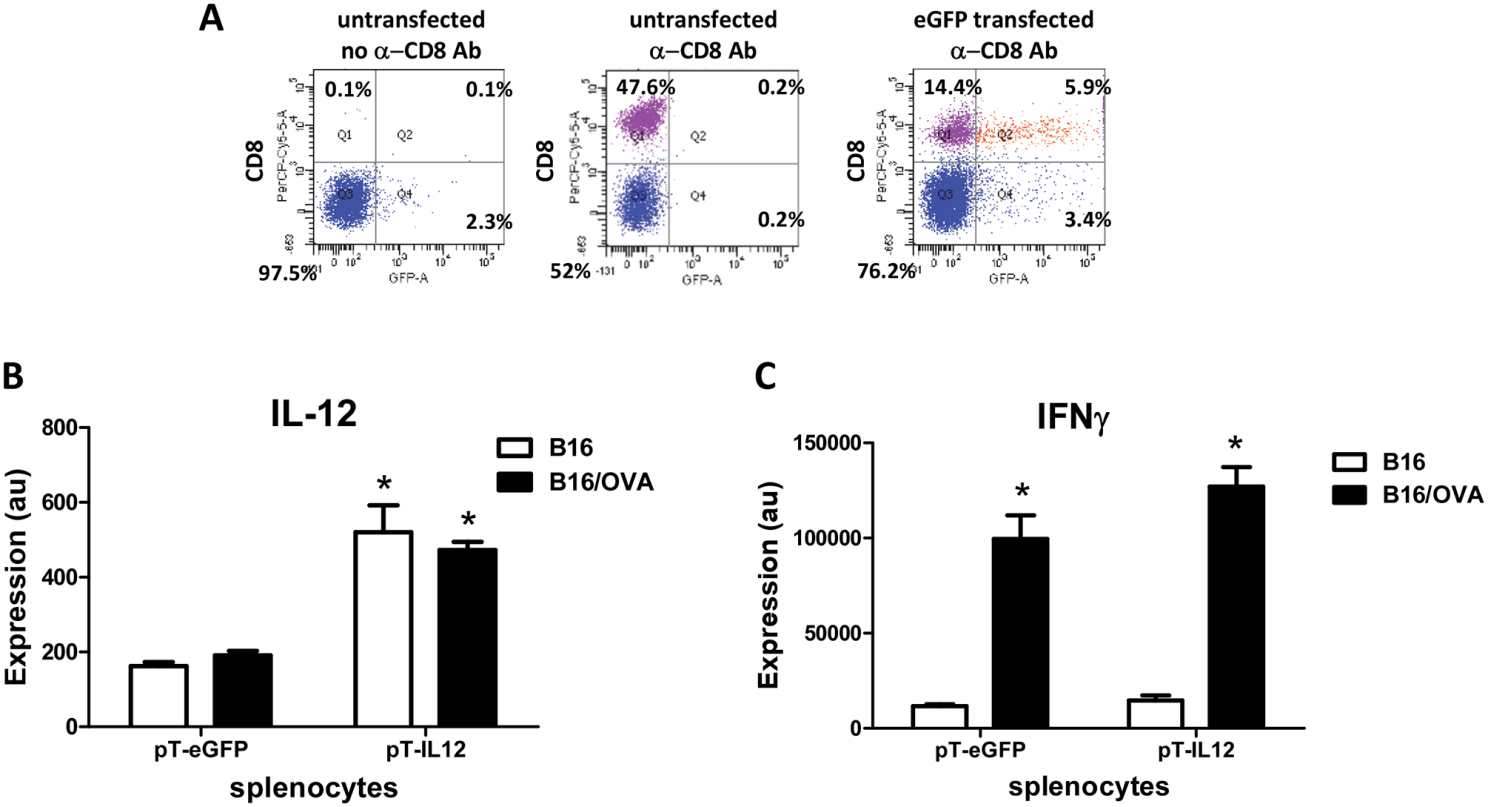
IL-12 transfected OT-I cells produce IL-12 and produce IFNγ when co-cultured with B16/OVA cells. **A**, OT-I splenocytes were transfected with pT-eGFP (control) or pT-mIL12 and co-cultured with B16 or B16/OVA cells. Flow cytometry confirmed the presence of eGFP expressing CD8 positive OT-I cells at the end of the co-culture. Shown is a representative of 3 independent experiments. **B**, cytometric bead analysis was used to measure mIL-12 (au, arbitrary units) in the media derived from the co-culture. *, p<0.05 comparing mIL-12 groups to eGFP. **C**, cytometric bead analysis was used to measure INFg production from transfected OT-I cells in the presence of B16 or B16/OVA cells. *, p<0.05 comparing B16/OVA groups to B16 (without OVA).

We performed co-culture experiments of gene modified OT-I cells with B16 cells to confirm mIL-12 expression from and antigen-specificity of the OT-I cells. OT-I cells were transfected with either pT-eGFP (control vector) or pT-mIL12 to produce mIL-12. Transfected OT-I splenocytes were then co-cultured with B16 or B16/OVA cells (Fig 5). Flow cytometry confirmed that 25 ± 3% of CD8 positive OT-I cells expressed eGFP (N = 3, ± STD) at the end of the co-culture (Fig 5A). Cytometric bead array analysis of media from the co-culture revealed increased mIL-12 (3.2 ± 0.9 fold when co-cultured with B16 and 2.5 ± 0.3 with B16/OVA; N = 3 ± STD) in pT-IL12 transfected splenocytes compared to eGFP controls (Fig 5B). To confirm antigen specificity, cytometric bead analysis of media from the co-culture for interferon-γ (INFγ) revealed increased INFγ (8.9 ± 3.3 fold for eGFP and 9.5 ± 4.4 fold for mIL-12 transfected splenocytes; N = 3 ± STD) when OT-I splenocytes were co-cultured with B16/OVA compared to B16 without OVA (Fig 5C). These results demonstrate mIL-12 expression from the pT-mIL12 vector from OT-I splenocytes in the presence of B16 or B16/OVA. Additionally, OT-I splenocytes increased INFγ production only when co-cultured with B16/OVA and this is unaffected by mIL-12 production from the OT-I splenocytes.

**Fig 6:**
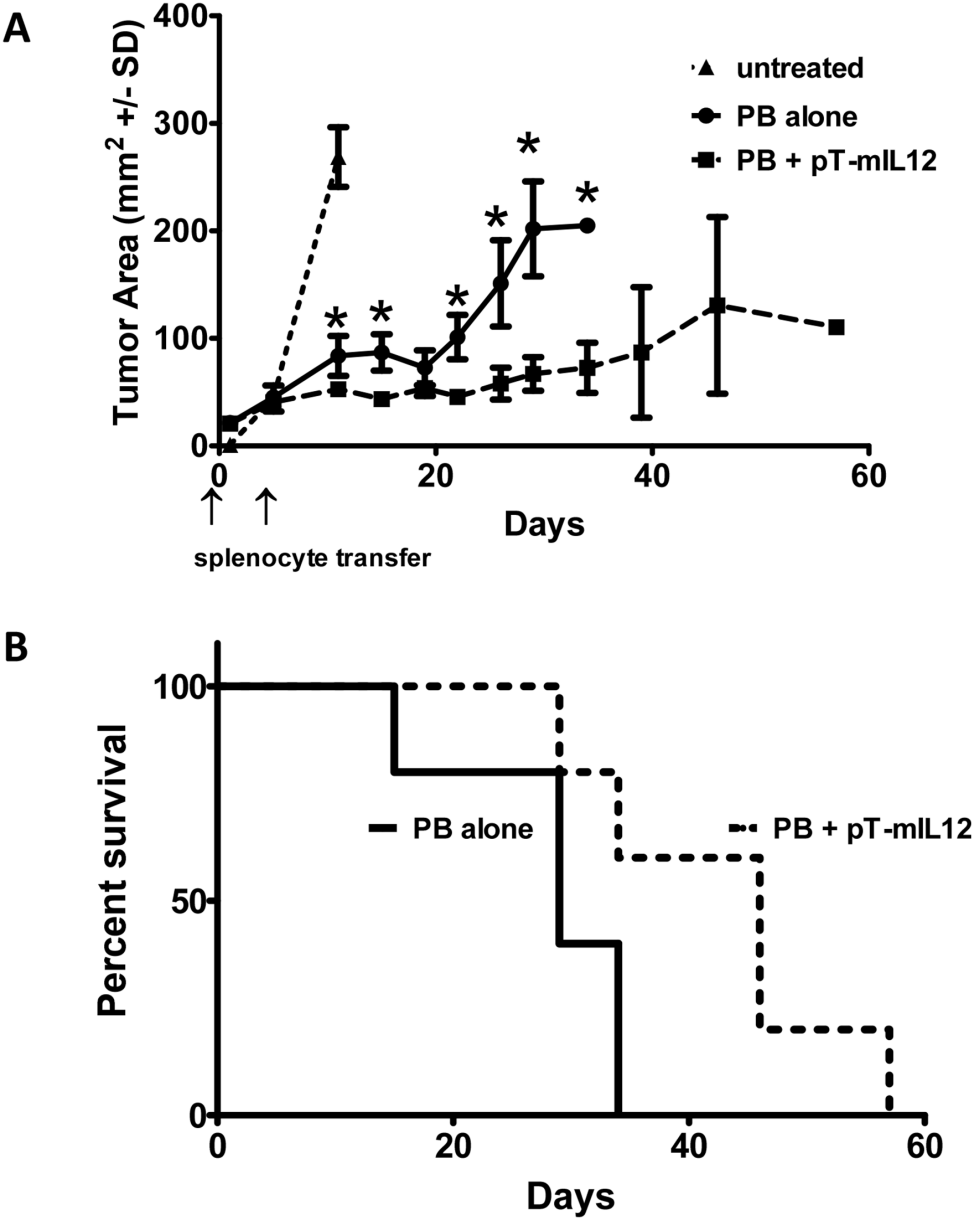
IL-12 *piggyBac*-modified mouse splenocytes exhibit anti-tumor activity *in vivo*. **A**, 5 X 10^5^ B16/OVA cells were transplanted into the flank of C57Bl6 mice.OT-I splenocytes modified with pT-mIL12 and pCMV-m7pB (compared to pCMV-m7pB alone) were adoptively transferred on day 0 and day 8 and tumor growth was monitored *in vivo* via caliper measurement of tumor diameter. *, p<0.05 using the student’s T test on the given day of comparison. OT-I splenocytes modified with pT-mIL12 slowed tumor growth *in vivo*. **B**, Adoptive transfer of *piggyBac* modified OT-1 splenocytes also improved mouse survival in the B16 melanoma model. The Mantel-Cox test exhibited a statistically different survival between the two groups, N = 10.

We next gene-modified mouse splenocytes with pT-IL12-Thy1.1 to evaluate the effect of mIL-12 on tumor growth *in vivo*. We implanted 5 X 10^5^ B16/OVA cells into the flank of 5 gray irradiated C57Bl6 mice. IL-12-modified OT-I splenocytes were adoptively transferred on day 0 and day 8 and tumor growth was monitored *in vivo*. Untreated, i.e. no adoptive transfer of OT-I splenocytes, mice exhibited rapid tumor growth (Fig 6A). Adoptive transfer of *piggyBac* alone modified splenocytes slowed tumor growth as expected given the antigen specificity of OT-I T cells directed towards the melanoma cells expressing the OVA antigen. Splenocytes modified with pT-mIL-12 slowed tumor growth *in vivo* even further when compared to OT-I cells without IL-12 (Fig 6A). Adoptive transfer of *piggyBac*-mIL-12 modified splenocytes also improved mouse survival in the B16 melanoma model (Fig 6B). Therefore, *piggyBac* modified mouse splenocytes expressing mIL-12 were capable of anti-tumor activity in an *in vivo* melanoma tumor model and had improved anti-tumor activity compared to splenocytes containing antigen specific T cells alone not expressing mIL-12.

## Materials and Methods

### *piggyBac* plasmid vectors

All *piggyBac* transposon vectors in the study were derived from zeo-pT-MCS [34]. We used the hyperactive pCMV-m7pB transposase plasmid [8, 9]. The mIL-12 construct used in this study was derived from pORF-mIL-12 from Invivogen (San Diego, CA). Standard molecular biology techniques were used to generate pT-mIL12-2A-venus, pT-mIL-12, pT-effLuc-Thy1.1 and pT-mIL12-Thy1.1 (Fig 1). All plasmids were prepared to be endotoxin free (Qiagen, Valencia, CA)The sequence of all plasmid was confirmed using DNA sequencing. Plasmids are available from the authors upon request.

### Cell culture and transfection

HeLa cells were cultured and transfected using FuGENE-6 as described previously [35]. Venus reporter gene expression was visualized in HeLa cells using a Leica fluorescent microscope. B16/OVA cells were cultured and transfected as described previously [21]. Secreted mIL-12 was quantitated via ELISA using an antibody to IL-12 (R & D Systems, Minneapolis, MN) and a Fluostar Omega microplate reader (BMG Labtech, Cary, NC).

### Splenocyte preparation, co-culture, and adoptive transfer

This research was approved by the IACUC of Baylor College of Medicine. Mouse splenocytes were prepared as described previously [21]. Briefly, the mouse spleen was removed and placed in sterile PBS. The spleen was ground and gently mashed through a 70μm mesh. Cells were pelleted by centrifugation (400 X g), layered over lympholyte (Cedarlane Labs, Burlington, NC), centrifuged (1000 X g) and the fuzzy and distinct layers were transferred to a fresh tube as described in according to the manufacturer’s protocol. Cells were washed twice in phosphate buffered saline (PBS), counted in 3% acetic acid, and 7.5 X 10^6^ cells were plated per well in a 12 well plate in RPMI media (Life Technologies, Grand Island, NY) containing mIL-2 (10ng/ml; eBioscience, San Diego, CA) and concanavalin-A (ConA, 5μg/ml; Sigma-Aldrich, St. Louis, MO). The following day cells were pelleted by centrifugation, washed once in PBS and resuspended in RPMI containing IL2 (5ng/ml) and IL-15 (5ng/ml) and transfected using the Neon transfection system (Life Technologies, Grand Island, NY). 6 X 10^6^ cells were transfected with a total of 10μg of DNA at a 1:3 ratio (transposase to transposon) at 1700V for two 15 ILlisecond pulses. After transfection, cells were plated in fresh media containing IL-2 and IL-15 as above.

For co-culture of transfected splenocytes with tumor cells, B16 or B16/OVA cells were seeded at 50,000 cells per 15.6mm well (standard 24 well plate) in 1ml of media (as described above but without ConA or IL2) an hour prior to transfection of splenocytes. After transfection, the 6 X 10^6^ cells were transferred to a single 15.6mm well containing B16 cells and 1ml of media. After 24 hours undiluted media was analyzed using a BD cytokine bead array (BD Biosciences, San Jose, CA) according to manufacturer’s instructions. Splenocytes were then obtained from each well by rinsing with media and analyzed by flow cytometry as described below.

Prior to adoptive transfer of IL-12 *piggyBac*-modified cells, the mice were irradiated with 5Gy of immunodepleting radiation as described by others [33]. The perpendicular diameter of tumors was measured by an independent investigator. Mice were sacrificed once the endpoint was reached as defined by the Baylor College of Medicine Institutional Animal Care and Use Committee. If a tumor reached 20mm, the mouse was euthanized. Animals were monitored 3 times per week and weighed 2 times per week until tumors reached 10mm. Once the tumor reached 10mm, the animal was monitored daily. Mice were euthanized via CO_2_ inhalation. Mice were euthanized if they appeared moribund, in pain or distress, exhibited inactivity, or loss 20% of pre-procedure body weight. The number and timing of cells infused for adoptive cell transfer was as described in the results section.

### Flow cytometry

Transfected splenocytes and mouse T cells were analyzed via flow cytometry using the following anitbodies: CD3, PerCP Hamster anti-mouse CD3e 145-2c11(BD Biosciences, San Jose, CA); Thy1.1, anti-mouse/rat CD90.1 (Thy1.1) PE (eBioscience); CD8, Anti-mouse CD8-PerCP (BD Biosciences). Expression was analyzed using a FACSCalibur flow cytometer with Cell Quest Software (Becton Dickinson, Franklin Lakes, NJ).

### Statistical analysis

Analysis between two groups was performed using the Student’s T test. Analysis between more than two groups was performed using two way ANOVA followed by Bonferroni posttests. Survival curves were compared using the Mantel-Cox test. P<0.05 was considered significant.

## Discussion

In order to determine if the *piggyBac* transposon system could be used to gene-modify mouse T cells for anti-tumor effects, we chose to modify splenocytes containing OVA antigen-specific OT-I T cells in the B16/OVA melanoma mouse model. We demonstrated *piggyBac* transposon mediated modification of murine T cells with reporter genes, a cell surface marker, and IL-12. Splenocytes modified with luciferase could home to tumor sites *in vivo*. Splenocytes containing antigen-specific T cells modified with IL-12 exhibited anti-tumor activity delaying both tumor growth and mortality in an animal model of melanoma.

Transposons have proven capability in gene modifying human T cells. Recently, the *sleeping beauty* transposon system has been approved for a clinical trial in gene modifying T cells to be directed to CD19 antigens [4–6]. While, immunodeficient mouse models (such as NOD-SCID), can be used to determine if antigen-specific T cells can traffic to and kill target cells *in vivo*, they are not useful for evaluation of genetic strategies designed to counteract tumor evasion strategies that involve a network of immune system cells. Further, human T cells cannot be used to evaluate adoptive T cell transfer for the treatment of naturally occurring or genetically induced tumors in animal models. Given the promising clinical potential of using transposons for immunotherapy applications [4, 36, 37], this study was undertaken to determine if transposons could gene-modify mouse T cells and target a mouse tumor *in vivo*.

Previous reports have demonstrated transfection of mouse T cells with plasmid DNA. Transposons offer the capability of permanently integrating transgene(s) into the mouse T cell genome with high efficiency. To determine if we could stably gene-modify mouse T cells and achieve anti-tumor activity *in vivo*, we chose IL-12 as a transgene because of its proven anti-tumor activity in previous reports. IL-12 is a TH1 cytokine, important for the TH1 polarization of T cells and dendritic cells that is essential to maintain effector T cell function. Previous studies have evaluated recombinant IL-12 therapy for cancer in humans, but dose-limiting toxicity resulted in limited efficacy [38–40]. More recent pre-clinical studies have focused on IL-12 production at tumor sites. Investigators have injected fibroblasts expressing IL-12 into tumors [28], electroporated IL-12 plasmids into metastatic melanoma lesions [41], and delivery of IL-12 via retrovirally transduced tumor antigen specific T cells [33, 42]. In this study, we used the non-viral *piggyBac* transposon system to gene-modify mouse splenocytes containing antigen-specific T cells to express IL-12 and demonstrated anti-tumor activity *in vivo*. Splenocytes harboring antigen-specific murine T cells could be gene modified with *piggyBac* and their homing to tumor sites could be visualized *in vivo*. IL-12 exhibited anti-tumor activity if expressed from B16 melanoma cells or from splenocytes containing antigen-specific (OT-I) T cells. Future experiments can be directed at testing other *piggyBac* modification of mouse T cells directed at specific melanoma antigens [43].

The recent successes and failures of CD19.CAR-modified human T-cells has demonstrated that numerous structural modification to the CAR backbone profoundly influence the expansion and persistence of CAR-T-cells after infusion [44, 45]. Further different methods of T-cell expansion and selection may further contribute to T-cell fate. The clinical implication of these differences cannot be modeled in vitro or in animal models and can only be tested in clinical trials. However, the cost of clinical grade viral vector production for the comparison of multiple constructs in differently produced T-cells is prohibitive and major economic commitments are frequently made for the generation of constructs with minimal clinical benefit. The use of inexpensive plasmids to test small modification would greatly enhance the progress of clinical trials and our understanding of the critical components of success. Using *piggyBac* to gene-modify mouse T cells is limited by transfection efficiency and the toxicity of the transfection methodology which reduces viable cells by as much as 50–80%; this holds true for human T cell modification as well. Additionally, *piggyBac* integrations are non-targeted which could result from undesired outcomes from genome modification. However, no genotoxic events have been observed thus far with non-targeted retroviral vectors when modifying human T cells in patients.

Our study demonstrates that splenocytes and mouse T cells can be gene modified with *piggyBac* for the testing of adoptive immunotherapy strategies in mice. Transposons could be used to compare the effect of multiple transgenes expressed in T cells or other cell types *in vivo*. We have recently demonstrated the ability of *piggyBac* to achieve multiplexed transposon modification of human cells [11]. Therefore, one could gene-modify mouse T cells with multiple transgenes such as tumor-directed chimeric antigen receptors, anti-tumor cytokines, or dominant negative receptors for inhibitors of T cell growth and function in animals with complete T cell repertoire and intact immune system thereby recapitulating the setting of adoptive cell transfer for immunotherapy in humans.

## Supporting Information

S1 Fig Standard curve for IL-12 ELISA. Serial dilutions of recombinant mIL-12 were used in an ELISA as described in the Materials and Methods section. Media dilutions were then compared to this standard curve for determination of the concentration of mIL-12 produced from transfected cells.
(TIF)

## References

[1] Dudley ME, Rosenberg SA. Adoptive-cell-transfer therapy for the treatment of patients with cancer. NatRevCancer. 2003;3(9):666–75. 10.1038/nrc1167 PMC2305722 12951585

[2] Rosenberg SA, Restifo NP, Yang JC, Morgan RA, Dudley ME. Adoptive cell transfer: a clinical path to effective cancer immunotherapy. NatRevCancer. 2008;8(4):299–308. 10.1038/nrc2355 PMC2553205 18354418

[3] Stromnes IM, Schmitt TM, Chapuis AG, Hingorani SR, Greenberg PD. Re-adapting T cells for cancer therapy: from mouse models to clinical trials. ImmunolRev. 2014;257(1):145–64. 10.1111/imr.12141 PMC4015625 24329795

[4] Hackett PB, Largaespada DA, Cooper LJ. A transposon and transposase system for human application. MolTher. 2010;18(4):674–83. 10.1038/mt.2010.2 PMC2862530 20104209

[5] Singh H, Manuri PR, Olivares S, Dara N, Dawson MJ, Huls H, et al Redirecting specificity of T-cell populations for CD19 using the Sleeping Beauty system. Cancer Res. 2008;68(8):2961–71. 10.1158/0008-5472.CAN-07-5600 18413766 PMC2424272

[6] Singh H, Huls H, Kebriaei P, Cooper LJ. A new approach to gene therapy using Sleeping Beauty to genetically modify clinical-grade T cells to target CD19. ImmunolRev. 2014;257(1):181–90. 10.1111/imr.12137 PMC4109051 24329797

[7] Vandendriessche T, Ivics Z, Izsvak Z, Chuah MK. Emerging potential of transposons for gene therapy and generation of induced pluripotent stem cells. Blood. 2009;114(8):1461–8. 10.1182/blood-2009-04-210427 19471016

[8] Doherty JE, Huye LE, Yusa K, Zhou L, Craig NL, Wilson MH. Hyperactive piggyBac Gene Transfer in Human Cells and In Vivo. Human Gene Therapy. 2012;23(3):311–20. 10.1089/hum.2011.138 21992617 PMC3300075

[9] Yusa K, Zhou L, Li MA, Bradley A, Craig NL. A hyperactive piggyBac transposase for mammalian applications. ProcNatlAcadSciUS A. 2011;108(4):1531–6. 10.1073/pnas.1008322108 PMC3029773 21205896

[10] Li MA, Turner DJ, Ning Z, Yusa K, Liang Q, Eckert S, et al Mobilization of giant piggyBac transposons in the mouse genome. Nucleic Acids Res. 2011;39(22):e148 10.1093/nar/gkr764 21948799 PMC3239208

[11] Kahlig KM, Saridey SK, Kaja A, Daniels MA, George AL Jr., Wilson MH. Multiplexed transposon-mediated stable gene transfer in human cells. Proceedings of the National Academy of Sciences of the United States of America. 2010;107(4):1343–8. 10.1073/pnas.0910383107 20080581 PMC2824351

[12] Fraser MJ, Ciszczon T, Elick T, Bauser C. Precise excision of TTAA-specific lepidopteran transposons piggyBac (IFP2) and tagalong (TFP3) from the baculovirus genome in cell lines from two species of Lepidoptera. Insect MolBiol. 1996;5(2):141–51. 10.1111/j.1365-2583.1996.tb00048.x 8673264

[13] Wilson MH, Coates CJ, George AL Jr. PiggyBac transposon-mediated gene transfer in human cells. Molecular Therapy. 2007;15(1):139–45. 17164785 10.1038/sj.mt.6300028

[14] Nakazawa Y, Saha S, Galvan DL, Huye L, Rollins L, Rooney CM, et al Evaluation of Long-term Transgene Expression in piggyBac-Modified Human T Lymphocytes. Journal of Immunotherapy. 2013;36(1):3–10. 10.1097/CJI.0b013e3182791234 23211626 PMC3521868

[15] Manuri PV, Wilson MH, Maiti SN, Mi T, Singh H, Olivares S, et al piggyBac transposon/transposase system to generate CD19-specific T cells for the treatment of B-lineage malignancies. HumGene Ther. 2010;21(4):427–37. 10.1089/hum.2009.114 PMC2938363 19905893

[16] Nakazawa Y, Huye LE, Dotti G, Foster AE, Vera JF, Manuri PR, et al Optimization of the PiggyBac transposon system for the sustained genetic modification of human T lymphocytes. JImmunother. 2009;32(8):826–36. 19752751 10.1097/CJI.0b013e3181ad762b PMC2796278

[17] Nakazawa Y, Huye LE, Salsman VS, Leen AM, Ahmed N, Rollins L, et al PiggyBac-mediated cancer immunotherapy using EBV-specific cytotoxic T-cells expressing HER2-specific chimeric antigen receptor. MolTher. 2011;19(12):2133–43. 10.1038/mt.2011.131 PMC3242651 21772253

[18] Goffinet C, Keppler OT. Efficient nonviral gene delivery into primary lymphocytes from rats and mice. FASEB J. 2006;20(3):500–2. 16401643 10.1096/fj.05-4651fje

[19] Gough M, Crittenden M, Thanarajasingam U, Sanchez-Perez L, Thompson J, Jevremovic D, et al Gene therapy to manipulate effector T cell trafficking to tumors for immunotherapy. JImmunol. 2005;174(9):5766–73. 15843579 10.4049/jimmunol.174.9.5766

[20] Kedl RM, Rees WA, Hildeman DA, Schaefer B, Mitchell T, Kappler J, et al T cells compete for access to antigen-bearing antigen-presenting cells. JExpMed. 2000;192(8):1105–13. 10.1084/jem.192.8.1105 PMC2195874 11034600

[21] Song XT, Turnis ME, Zhou X, Zhu W, Hong BX, Rollins L, et al A Th1-inducing adenoviral vaccine for boosting adoptively transferred T cells. MolTher. 2011;19(1):211–7. 10.1038/mt.2010.223 PMC3017450 20959814

[22] Dudley ME, Wunderlich JR, Yang JC, Sherry RM, Topalian SL, Restifo NP, et al Adoptive cell transfer therapy following non-myeloablative but lymphodepleting chemotherapy for the treatment of patients with refractory metastatic melanoma. JClinOncol. 2005;23(10):2346–57. 10.1200/JCO.2005.00.240 PMC1475951 15800326

[23] Dudley ME, Yang JC, Sherry R, Hughes MS, Royal R, Kammula U, et al Adoptive cell therapy for patients with metastatic melanoma: evaluation of intensive myeloablative chemoradiation preparative regimens. JClinOncol. 2008;26(32):5233–9. 10.1200/JCO.2008.16.5449 PMC2652090 18809613

[24] Morgan RA, Dudley ME, Wunderlich JR, Hughes MS, Yang JC, Sherry RM, et al Cancer regression in patients after transfer of genetically engineered lymphocytes. Science. 2006;314(5796):126–9. 16946036 10.1126/science.1129003 PMC2267026

[25] Gately MK. Interleukin-12: a recently discovered cytokine with potential for enhancing cell-mediated immune responses to tumors. Cancer Invest. 1993;11(4):500–6. 8100734 10.3109/07357909309018881

[26] Mehrotra PT, Wu D, Crim JA, Mostowski HS, Siegel JP. Effects of IL-12 on the generation of cytotoxic activity in human CD8+ T lymphocytes. JImmunol. 1993;151(5):2444–52. 8103066

[27] Chinnasamy D, Yu Z, Kerkar SP, Zhang L, Morgan RA, Restifo NP, et al Local delivery of interleukin-12 using T cells targeting VEGF receptor-2 eradicates multiple vascularized tumors in mice. ClinCancer Res. 2012;18(6):1672–83. 10.1158/1078-0432.CCR-11-3050 PMC6390958 22291136

[28] Kang WK, Park C, Yoon HL, Kim WS, Yoon SS, Lee MH, et al Interleukin 12 gene therapy of cancer by peritumoral injection of transduced autologous fibroblasts: outcome of a phase I study. HumGene Ther. 2001;12(6):671–84. 10.1089/104303401300057388 11426466

[29] Zitvogel L, Tahara H, Robbins PD, Storkus WJ, Clarke MR, Nalesnik MA, et al Cancer immunotherapy of established tumors with IL-12. Effective delivery by genetically engineered fibroblasts. JImmunol. 1995;155(3):1393–403. 7636204

[30] Lucas ML, Heller L, Coppola D, Heller R. IL-12 plasmid delivery by in vivo electroporation for the successful treatment of established subcutaneous B16.F10 melanoma. MolTher. 2002;5(6):668–75. 10.1006/mthe.2002.0601 12027550

[31] Teicher BA, Ara G, Buxton D, Leonard J, Schaub RG. Optimal scheduling of interleukin-12 and fractionated radiation therapy in the murine Lewis lung carcinoma. RadiatOncolInvestig. 1998;6(2):71–80. 10.1002/(SICI)1520-6823(1998)6:2<71::AID-ROI2>3.0.CO;2-E 9572683

[32] Basile LA, Ellefson D, Gluzman-Poltorak Z, Junes-Gill K, Mar V, Mendonca S, et al HemaMax, a recombinant human interleukin-12, is a potent mitigator of acute radiation injury in mice and non-human primates. PLoSOne. 2012;7(2):e30434. 10.1371/journal.pone.0030434 PMC3286478 22383962

[33] Zhang L, Kerkar SP, Yu Z, Zheng Z, Yang S, Restifo NP, et al Improving adoptive T cell therapy by targeting and controlling IL-12 expression to the tumor environment. MolTher. 2011;19(4):751–9. 10.1038/mt.2010.313 PMC3070103 21285960

[34] Nakazawa Y, Saha S, Galvan DL, Huye L, Rollins L, Rooney CM, et al Evaluation of long-term transgene expression in piggyBac-modified human T lymphocytes. JImmunother. 2013;36(1):3–10. 23211626 10.1097/CJI.0b013e3182791234 PMC3521868

[35] Wilson MH, Coates CJ, George AL Jr. PiggyBac Transposon-mediated Gene Transfer in Human Cells. MolTher. 2007;15(1):139–45. 10.1038/sj.mt.6300028 17164785

[36] Hackett PB, Largaespada DA, Switzer KC, Cooper LJ. Evaluating risks of insertional mutagenesis by DNA transposons in gene therapy. TranslRes. 2013;161(4):265–83. 10.1016/j.trsl.2012.12.005 PMC3602164 23313630

[37] Izsvak Z, Hackett PB, Cooper LJ, Ivics Z. Translating Sleeping Beauty transposition into cellular therapies: victories and challenges. Bioessays. 2010;32(9):756–67. 10.1002/bies.201000027 20652893 PMC3971908

[38] Atkins MB, Robertson MJ, Gordon M, Lotze MT, DeCoste M, DuBois JS, et al Phase I evaluation of intravenous recombinant human interleukin 12 in patients with advanced malignancies. ClinCancer Res. 1997;3(3):409–17. 9815699

[39] Gollob JA, Mier JW, Atkins MB. Clinical use of systemic IL-12 therapy. Cancer ChemotherBiolResponse Modif. 2001;19:353–69. 11686023

[40] Leonard JP, Sherman ML, Fisher GL, Buchanan LJ, Larsen G, Atkins MB, et al Effects of single-dose interleukin-12 exposure on interleukin-12-associated toxicity and interferon-gamma production. Blood. 1997;90(7):2541–8. 9326219

[41] Daud AI, DeConti RC, Andrews S, Urbas P, Riker AI, Sondak VK, et al Phase I trial of interleukin-12 plasmid electroporation in patients with metastatic melanoma. JClinOncol. 2008;26(36):5896–903. 10.1200/JCO.2007.15.6794 PMC2645111 19029422

[42] Kerkar SP, Muranski P, Kaiser A, Boni A, Sanchez-Perez L, Yu Z, et al Tumor-specific CD8+ T cells expressing interleukin-12 eradicate established cancers in lymphodepleted hosts. Cancer Res. 2010;70(17):6725–34. 10.1158/0008-5472.CAN-10-0735 20647327 PMC2935308

[43] Overwijk WW, Tsung A, Irvine KR, Parkhurst MR, Goletz TJ, Tsung K, et al gp100/pmel 17 is a murine tumor rejection antigen: induction of "self"-reactive, tumoricidal T cells using high-affinity, altered peptide ligand. JExpMed. 1998;188(2):277–86. 10.1084/jem.188.2.277 PMC2212458 9670040

[44] Hudecek M, Sommermeyer D, Kosasih PL, Silva-Benedict A, Liu L, Rader C, et al The nonsignaling extracellular spacer domain of chimeric antigen receptors is decisive for in vivo antitumor activity. Cancer Immunol Res. 2015;3(2):125–35. 10.1158/2326-6066.CIR-14-0127 25212991 PMC4692801

[45] Jensen MC, Riddell SR. Designing chimeric antigen receptors to effectively and safely target tumors. Curr Opin Immunol. 2015;33:9–15. 10.1016/j.coi.2015.01.002 25621840 PMC4397136

