# Supplementary figures and images for: Anti-Tumor Effects after Adoptive Transfer of IL-12 Transposon-Modified Murine Splenocytes in the OT-I-Melanoma Mouse Model
Source: PLoS One. 2015 Oct 16;10(10):e0140744. doi: 10.1371/journal.pone.0140744 (PMC4608718; doi:10.1371/journal.pone.0140744)

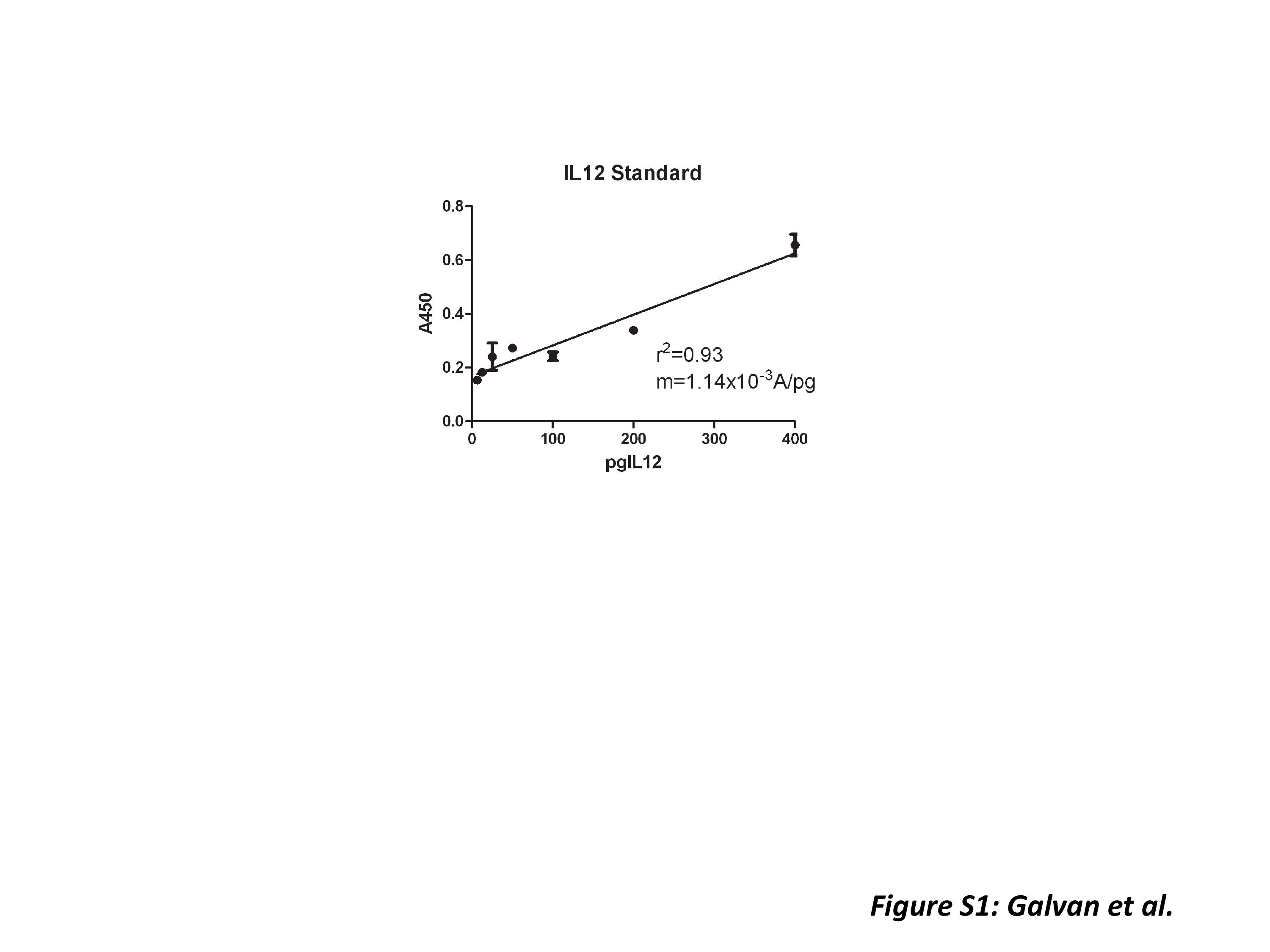

Supplement: S1 Fig — Serial dilutions of recombinant mIL-12 were used in an ELISA as described in the Materials and Methods section. Media dilutions were then compared to this standard curve for determination of the concentration of mIL-12 produced from transfected cells. (TIF) [file pone.0140744.s001.tif]
